# Prognostic value of Musashi 2 (MSI2) in cancer patients: A systematic review and meta-analysis

**DOI:** 10.3389/fonc.2022.969632

**Published:** 2022-12-01

**Authors:** Lin Jiang, Shanshan Xue, Jie Xu, Xiaoyang Fu, Jing Wei, Chuanmeng Zhang

**Affiliations:** ^1^ Department of Anesthesiology, Taizhou People’s Hospital, Affiliated to Nanjing Medical University, Taizhou, Jiangsu, China; ^2^ Department of Clinical Laboratory, Taizhou People’s Hospital, Affiliated to Nanjing Medical University, Taizhou, Jiangsu, China; ^3^ The Center for Translational Medicine, Taizhou People’s Hospital, Affiliated to Nanjing Medical University, Taizhou, Jiangsu, China; ^4^ Department of Obstetrics and Gynecology, Taizhou People’s Hospital, Affiliated to Nanjing Medical University, Taizhou, Jiangsu, China

**Keywords:** MSI2, meta-analysis, prognosis, clinicopathological features, cancer

## Abstract

Musashi 2 (MSI2) is an RNA-binding protein that regulates mRNA translation of numerous intracellular targets and plays an important role in the development of cancer. However, the prognostic value of MSI2 in various cancers remains controversial. Herein, we conducted this meta-analysis including 21 studies with 2640 patients searched from PubMed, Web of Science, EMBASE, Chinese National Knowledge Infrastructure databases, and WanFang databases to accurately assess the prognostic significance of MSI2 in various cancers. Our results indicated that high MSI2 expression was significantly related to poor overall survival (HR = 1.84, 95% CI: 1.66-2.05, *P* < 0.001) and disease-free survival (HR = 1.73, 95% CI: 1.35-2.22, *P* < 0.001). In addition, MSI2 positive expression was associated with certain phenotypes of tumor aggressiveness, such as clinical stage, depth of invasion, lymph node metastasis, liver metastasis and tumor size. In conclusion, elevated MSI2 expression is closely correlated with poor prognosis in various cancers, and may serve as a potential molecular target for cancer patients.

## Introduction

According to recently released data, there would be 19.29 million new cancer cases and 9.96 million deaths worldwide in 2020, which remains a global and growing public health problem (1). Although targeted therapy and comprehensive treatment for cancers have made remarkable progress, the therapeutic effect of most tumors is still unsatisfactory (2). The main reason is the lack of effective methods for prognosis monitoring of cancer patients (3). Thus, identification of new biomarkers with the potential to predict cancer progression and prognosis will bring new hope to cancer patients.

Posttranscriptional regulation is known to control gene expression and cell behavior (4). Accumulating evidence indicates that aberrant expression and dysfunction of RNA-binding proteins (RBPs) as posttranscriptional regulators are associated with initiation, progression, and chemoresistance of various types of tumors (5, 6). The RBP Musashi-2 (MSI2) has been characterized as a cancer-driver gene in some cancers (7). It binds and regulates the mRNA stability and translation of proteins operating in vital oncogenic signaling pathways, including NUMB/Notch, PTEN/Akt/mTOR, TGFβ/SMAD, MYC, cMET, and others (7, 8). In pancreatic cancer, Sheng et al. revealed that Msi2 promotes the occurrence and development of pancreatic cancer by downregulating Numb protein that can regulate various carcinogenic signaling pathways, including Notch, p53 and Hedgehog pathways (9). Wang et al. found that Msi2 can inhibit tumor suppressor gene PTEN and activate PDK/Akt/mTORC1 signal pathway to cause tumor (10). Jiang et al. showed that Msi2 expression regulates epithelial to mesenchymal transition (EMT) by activating transcription factors Snail and TGFβR1/Smad3 signaling, which is related to chemoresistency of glioblastoma (11). Moreover, TGFβ/Smad signaling pathway is involved in cell proliferation, differentiation, apoptosis, adhesion, invasion and cell microenvironment (8, 12). In addition, multiple other studies also showed that MSI2 protein maintains cancer stem cell populations and regulates cancer invasion, metastasis and development of more aggressive cancer phenotypes, including drug resistance (8, 13–18). Thus, MSI2 seems to be a potential prognostic biomarker and therapeutic target for cancer patients.

MSI2 has been proved to be significantly up-regulated in various cancers, such as ovarian carcinoma (OC) (19), non-small cell lung cancer (NSCLC) (20), colorectal cancer (CRC) (21), cervical cancer (CC) (16), et al. Moreover, excessive MSI2 expression is associated with poor prognosis in numerous solid tumors as well as in hematological malignancies (9, 15, 19–39), but the results are controversial (16, 32, 35, 36, 40, 41). Hence, we carried out this meta-analysis to further analyze the prognostic value of MSI2, so as to provide a theoretical basis for the prognosis and treatment of patients with cancer.

## Materials and methods

### Literature search

A systematic literature search of PubMed, Web of Science, EMBASE, CNKI, and Wanfang was performed through April 2022 to identify relevant papers reporting the association between MSI2 expression and survival outcomes (including overall survival [OS] and disease-free survival [DFS]) in patients with cancer. The following keywords were applied in the search: (“cancer” OR “neoplasm” OR “tumor” OR “carcinoma”) AND (“Musashi 2” OR “MSI2”) AND (“prognosis” OR “survival” OR “mortality”). An additional manual search of references cited in eligible articles was also conducted to ensure that all relevant studies were included.

### Inclusion and exclusion criteria

The inclusion criteria were as follows: articles that assess the association between MSI2 expression and prognosis in patients with cancer; hazard ratio (HR) and 95% confidence interval (CI) that are provided directly or calculated with sufficient data; the expression of MSI2 in tumor tissues that are measured by immunohistochemistry (IHC), or quantitative reverse transcription polymerase chain reaction (qRT-PCR); patients that were divided into two groups according to MSI2 expression level.

The exclusion criteria were as follows: reviews, case reports, letters, and conference abstracts, etc; duplicated publication; or studies without sufficient data.

### Data extraction and quality assessment

Two authors independently extracted basic data and any difference was resolved through discussion until consensus was reached. The basic information is as follows: the first author, publication year, country, duration time, cancer type, follow-up time, sample size, detection method, cut-off value, clinicopathological feature, clinical outcome, analysis method, and HR with corresponding 95% CI. For studies reporting HR values in univariate and multivariate analyses, we tend to choose the latter because of higher accuracy after adjusting for confounding factors. For articles only reporting the survival curve of OS or DFS, we estimated an HR value from the survival curve.

Two investigators independently evaluated the quality of the included articles using the Newcastle-Ottawa Scale (NOS) within the following domains: selection, 0-4; comparability, 0-2; and outcome, 0-3 (42). NOS score ≥6 was regarded as high quality (43).

### Statistical analysis

Stata software version 12.0 (StataCorp, College Station, TX) was used for all statistical analyses. HRs and 95% CIs were combined to evaluate the effect of MSI2 expression on prognosis. ORs and 95% CIs were pooled to assess the association of MSI2 expression with clinicopathological characteristics. Heterogeneity across studies was measured by the Chi Squared-based Q test and *I* ^2^ statistics. When *P*  < 0 .05 or *I* ^2^ >  50% indicated statistically significant heterogeneity between the studies, the random-effects model was applied for analysis. Otherwise, the fixed-effects model was used. Subgroup analysis was conducted to comprehensively evaluate the correlation between MSI2 expression and OS. Sensitivity analysis was carried out by removing one cohort at a time to prove the stability of the results. Potential publication bias was quantitatively evaluated through Begg’s and Egger’s tests and visually evaluated using funnel plots. The *P*  < 0.05 was considered to be statistically significant.

## Results

### Literature search and study demographics

**Figure 1:**
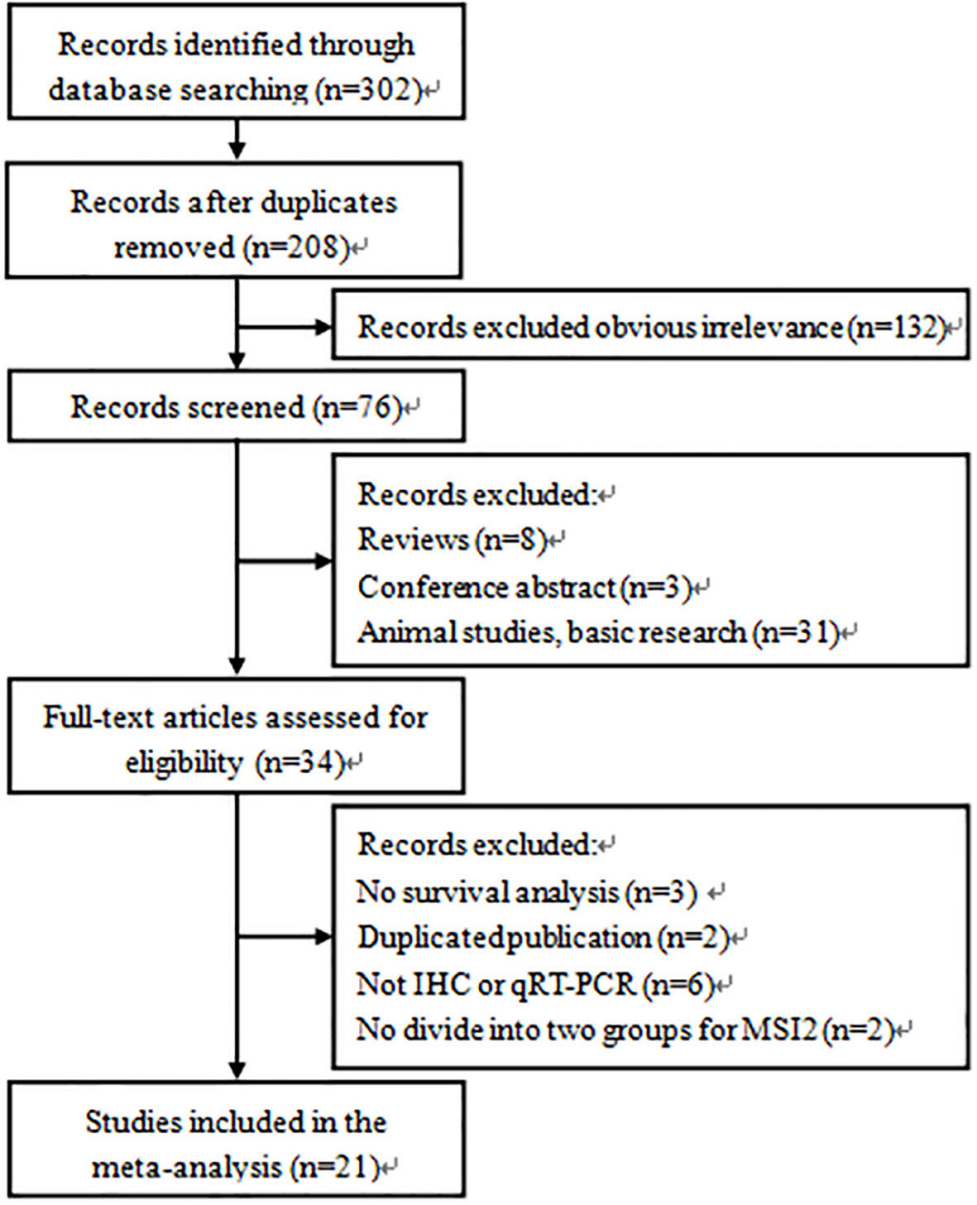
Flow diagram of the study selection process and specific reasons for exclusion of the studies in the meta-analysis.

A total of 302 applicable records were initially identified through the database search. After removing duplicate (n=94) and obvious irrelevance (n=132) articles, 76 studies were further evaluated by scanning titles and abstracts. Then, the remaining 34 studies were further evaluated by browsing full texts. Finally, 21 articles with 2640 patients were included in the meta-analysis. The flow chart of literature search and screening process was shown in **Figure 1**.

The characteristics of the included studies are summarized in **Table 1**. The articles included in this study were mainly from China (19, 21–30, 32–34, 36), and the rest were from Russia (16, 20), Italy (40), Egypt (31), Germany (35) and the UK (37), and were published from 2011 to 2022. The types of cancers in the enrolled studies were OC (19), NSCLC (20), CRC (16, 21, 26, 28), cervical cancer (CC) (22, 25), pancreatic cancer (PC) (23, 34), hepatocellular carcinoma (HCC) (24, 33), oral squamous cells carcinoma (OSCC) (40), esophageal squamous cell carcinoma (ESCC) (27), acute lymphoblastic leukemia (ALL) (29, 36), gastric carcinomas (GC) (30), and acute myeloid leukemia (AML) (31, 32, 35, 37). The expression levels of MSI2 were detected either by IHC (16, 19–26, 28, 33, 34, 37, 40) or by qRT-PCR (27, 29–32, 35, 36). OS (16, 19–37, 40) and DFS (24, 27, 31–33, 35, 36) were reckoned as survival outcomes. Based on NOS score, each study received a score of ≥ 6, indicating that the quality of all included studies was high.

**Table 1:** Main characteristics of the eligible studies.

Study	Region	Duration	Cancer type	Clinical stage	Follow up (months)	Number	Detection method	Cut-off value	Survival analysis	Language	Quality
Zhen J 2022	China	2010-2021	OC	I-IV	60	75	IHC	≥3	OS(M)	Chinese	8
Topchu I 2021	Russia	NR	NSCLC	I-IV	NR	40	IHC	Median	OS(U)	English	6
Li Y 2021	China	2015-2017	CRC	NR	36	180	IHC	≥4	OS(U)	Chinese	6
Kharin L 2021	Russia	NR	CRC	I-IV	NR	105	IHC	Median	OS(U)	English	6
Zhen J 2021	China	2012-2019	CC	I-IV	60	126	IHC	≥3	OS(M)	Chinese	8
Zhou L 2020	China	2006-2017	PC	I-IV	NR	91	IHC	>4	OS(M)	English	8
Wang X 2019	China	NR	HCC	I-IV	NR	82	IHC	≥6	OS(U); DFS(U)	English	6
Troiano G 2019	Italy	1997-2012	OSCC	I-IV	NR	108	IHC	NR	OS(M)	English	7
Liu Y 2018	China	2003-2007	CC	I-II	60	162	IHC	>4	OS(M)	English	7
Shen W 2017	China	2012-2016	CRC	NR	NR	85	IHC	>4	OS(U)	Chinese	6
Li Z 2017	China	NR	ESCC	I-IV	NR	62	qRT-PCR	Median	OS(U); DFS(U)	English	8
Zong Z 2016	China	2007-2012	CRC	I-IV	NR	164	IHC	>1.5	OS(M)	English	8
Zhao HZ 2016	China	2007-2010	ALL	NR	Median 67.5	119	qRT-PCR	75^th^	OS(M)	English	7
Yang Z 2016	China	2012	GC	I-IV	Mean 26.16	67	qRT-PCR	≥2	OS(U)	Chinese	6
Aly RM 2015	Egypt	2011-2014	AML	NR	NR	118	qRT-PCR	2.4	OS(M); DFS(M)	English	8
Lu Y 2014	China	2008-2012	AML	M_0_-M_4_	Until Oct 2013	181	qRT-PCR	median	OS(U); DFS(U)	Chinese	6
He L 2014	China	2005-2010	HCC	I-IV	Until Sep 2012	149	IHC	>1.5	OS(M); DFS(M)	English	8
Gao Z 2014	China	2005-2013	PC	I-III	NR	51	IHC	≥4	OS(U)	Chinese	
Thol F 2013	Germany	NR	AML	M_0_-M_7_	NR	454	qRT-PCR	75^th^	OS(M); DFS(U)	English	8/6
Mu Q 2013	China	2000-2010	ALL	NR	Until Mar 2012	101	qRT-PCR	75^th^	OS(U); DFS(U)	English	6
Byers RJ 2011	UK	1994-2005	AML	M_0_-M_7_	168	120	IHC	75^th^	OS(M)	English	8

OC, ovarian carcinoma; NSCLC, non-small cell lung cancer; CRC, colorectal cancer; CC, cervical cancer; PC, pancreatic cancer; HCC, hepatocellular carcinoma; OSCC, Oral Squamous Cells Carcinoma; ESCC, esophageal squamous cell carcinoma; ALL, acute lymphoblastic leukemia; GC, gastric carcinomas; AML, acute myeloid leukemia; IHC, immunohistochemistry; qRT-PCR, quantitative reverse transcription polymerase chain reaction; OS overall survival; DFS, disease-free survival; M, multivariate analysis; U, univariate analysis; NR, none reported.

### Association between MSI2 expression and prognosis

**Figure 2:**
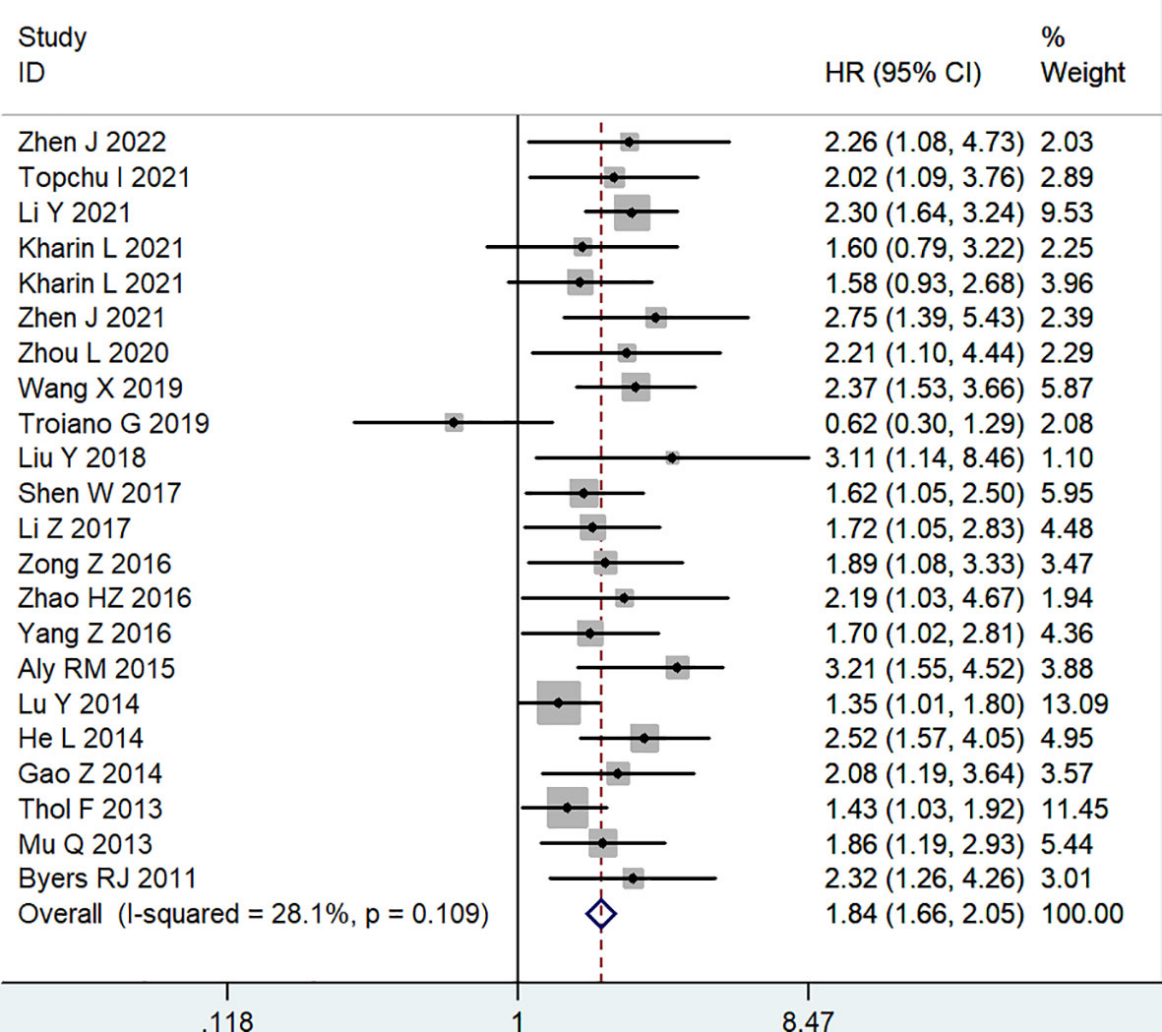
Forest plot of studies evaluating hazard ratios of high MSI2 expression and the overall survival of cancer patients.

All included studies reported OS to assess the association between MSI2 expression and prognosis. A fixed effects model was applied to calculate the combined HR (95% CI) due to the absence of significant heterogeneity (*I* ^2^ = 28.1%, *P* = 0.109). The results demonstrated that high expression levels of MSI2 were significantly associated with poorer OS in human cancers (HR = 1.84, 95% CI: 1.66-2.05, *P* < 0.001) (**Table 2**; **Figure 2**).

**Table 2:** Summary of the meta-analysis results.

Categories	Trials	HR (95%CI)	*I^2^ (%)*	*P_h_*	*Z*	*P*
OS (All) (16, 19–37, 40)	21 (2640)	1.84 (1.66-2.05)	28.1	0.109	11.36	<0.001
Cancer type						
Solid tumor (16, 19–28, 30, 33, 34, 40)	15 (1547)	1.96 (1.71-2.24)	9.4	0.346	9.78	<0.001
CRC (16, 21, 26, 28)	4 (534)	1.88 (1.53-2.32)	0.0	0.660	5.90	<0.001
CC (22, 25)	2 (288)	2.86 (1.63-5.02)	0.0	0.844	3.65	<0.001
PC (23, 34)	2 (142)	2.13 (1.38-3.29)	0.0	0.893	3.41	0.001
HCC (24, 33)	2 (231)	2.44 (1.77-3.36)	0.0	0.849	5.46	<0.001
Others (19, 20, 27, 30, 40)	5 (352)	1.60 (1.23-2.08)	49.9	0.092	3.48	0.001
Blood tumor (29, 31, 32, 35–37)	6 (1093)	1.83 (1.40-2.39)^R^	52.9	0.060	4.43	<0.001
ALL (29, 36)	2 (220)	1.94 (1.32-2.86)	0.0	0.718	3.36	0.001
AML (31, 32, 35, 37)	4 (873)	1.82 (1.26-2.63)^R^	69.3	0.021	3.19	0.001
Detection method						
IHC (16, 19–26, 28, 33, 34, 37, 40)	14 (1538)	2.02 (1.75-2.33)	13.1	0.307	9.73	<0.001
qRT-PCR (27, 29–32, 35, 36)	7 (1102)	1.64 (1.40-1.92)	36.6	0.146	6.17	<0.001
Sample size						
≥120 (21, 22, 25, 28, 32, 33, 35, 37)	8 (1536)	1.81 (1.55-2.10)	46.9	0.068	7.69	<0.001
<120 (16, 19, 20, 23, 24, 26, 27, 29–31, 34, 36, 40)	13 (1104)	1.88 (1.62-2.18)	18.3	0.254	8.37	<0.001
Analysis method						
Multivariate (19, 22, 23, 25, 28, 29, 31, 33, 35, 37, 40)	11 (1686)	2.04 (1.57-2.63)^R^	50.5	0.027	5.41	<0.001
Univariate (16, 20, 21, 24, 26, 27, 30, 32, 34, 36)	10 (954)	1.78 (1.56-2.04)	0.0	0.592	8.41	<0.001
DFS (All) (24, 27, 31–33, 35, 36)	7 (1147)	1.73 (1.35-2.22)^R^	66.2	0.007	4.28	<0.001

CRC, colorectal cancer; CC, cervical cancer; PC, pancreatic cancer; HCC, hepatocellular carcinoma; ALL, acute lymphoblastic leukemia; AML, acute myeloid leukemia; IHC, immunohistochemistry; qRT-PCR, quantitative reverse transcription polymerase chain reaction; OS overall survival; DFS, disease-free survival; HR, hazard ratio; CI, confidence interval; P_h_, P value for heterogeneity based on Q test; P, P value for statistical significance based on Z test.

Subgroup analyses were performed according to cancer type, detection method, sample size and analysis method to comprehensively evaluate the correlation between MSI2 expression and OS (**Table 2**). Subgroup analysis by cancer type showed that increased expression of MSI2 was significantly related to shorter OS in patients with solid tumors (HR = 1.96, 95% CI: 1.71-2.24, *P* < 0.001) (including CRC (HR = 1.88, 95% CI: 1.53-2.32, *P* < 0.001), CC (HR = 2.86, 95% CI: 1.63-5.02, *P* < 0.001), PC (HR = 2.13, 95% CI: 1.38-3.29, *P* = 0.001), HCC (HR = 2.44, 95% CI: 1.77-3.36, *P* < 0.001) and Others (HR = 1.60, 95% CI: 1.23-2.08, *P* = 0.001)) and blood tumors (HR = 1.83, 95% CI: 1.40-2.39, *P* < 0.001) (including ALL (HR = 1.94, 95% CI: 1.32-2.86, *P* = 0.001) and AML (HR = 1.82, 95% CI: 1.26-2.63, *P* = 0.001)). This result was similar to that obtained by subgroup analysis of detection method, such as IHC (HR = 2.02, 95% CI: 1.75-2.33, *P* < 0.001) and qRT-PCR (HR = 1.64, 95% CI: 1.40-1.92, *P* < 0.001). In addition, the association between high expression of MSI2 and poor OS was also detected in large (HR = 1.81, 95% CI: 1.55-2.10, *P* < 0.001) and small (HR = 1.88, 95% CI: 1.62-2.18, *P* < 0.001) subgroups. What is more, MSI2 overexpression was associated with poor OS in both multivariate (HR = 2.04, 95% CI: 1.57-2.63, *P* < 0.001) and univariate (HR = 1.78, 95% CI: 1.56-2.04, *P* < 0.001) subgroups.

**Figure 3:**
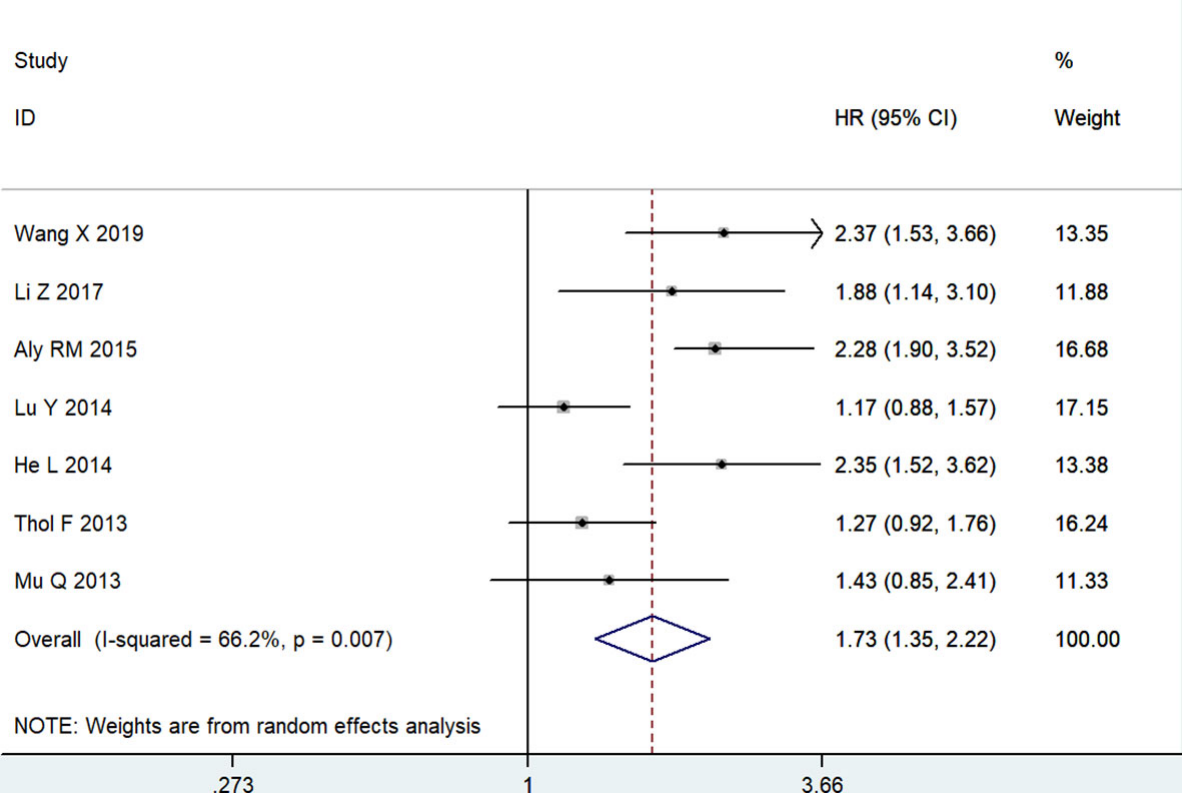
Forest plot of studies evaluating hazard ratios of high MSI2 expression and the disease-free survival of cancer patients.

Meanwhile, seven articles, including 1147 patients, evaluated the correlation between MSI2 expression and DFS. Due to significant heterogeneity among studies (*I* ^2^ = 66.2%, *P* = 0.007), a random effects model was employed to estimate the pooled HR and 95% CI of DFS. The pooled HR (HR = 1.73, 95% CI: 1.35-2.22, *P* < 0.001) showed that high MSI2 expression was significantly correlated with poorer DFS in patients with cancer (**Figure 3**).

### Association between MSI2 expression and clinicopathological features

To systematically analyze the role of MSI2 expression as a biomarker in cancer, we explored the relationship between MSI2 expression and clinicopathological features (**Table 3**). Six studies with 619 patients described the MSI2 expression and clinical stage, and the combined result demonstrated that high expression of MSI2 was obviously associated with advanced clinical stage (OR = 2.14, 95% CI: 1.19-3.85, *P* = 0.011). Moreover, this significant correlation was also observed in terms of depth of invasion (OR = 2.44, 95% CI: 1.65-3.61, *P* < 0.001), lymph node metastasis (OR = 2.60, 95% CI: 1.75-3.85, *P* < 0.001), liver metastasis (OR = 2.16, 95% CI: 1.31-3.54, *P* = 0.002) and tumor size (OR = 1.96, 95% CI: 1.16-3.31, *P* = 0.013). However, MSI2 expression had no significant association with age (OR = 1.07, 95% CI: 0.86-1.34, *P* = 0.549), gender (OR = 0.99, 95% CI: 0.79-1.22, *P* = 0.899) and degree of differentiation (OR = 0.68, 95% CI: 0.27-1.72, *P* = 0.418).

**Table 3:** Meta-analysis of MSI2 and clinicopathological features in cancer patients.

Categories	Trials (Patients)	OR (95%CI)	*I^2^* (*%*)	*P_h_*	*Z*	*P*
Age (young vs. old) (16, 19, 21, 22, 24, 25, 27, 28, 30, 32–34)	12 (1414)	1.07 (0.86-1.34)^F^	7.7	0.370	0.60	0.549
Gender (male vs. female) (16, 21, 27, 28, 30–32, 34–37)	11 (1650)	0.99 (0.79-1.22)^F^	0.0	0.885	0.13	0.899
clinical stage (I-II vs. III-IV) (16, 19, 22, 24, 28, 30)	6 (619)	2.14 (1.19-3.85)	61.7	0.023	2.53	0.011
Depth of invasion (T1-T2 vs. T3-T4) (16, 21, 26, 28, 30, 34)	6 (662)	2.44 (1.65-3.61)^F^	12.2	0.337	4.48	<0.001
Lymph node metastasis (negative vs. positive) (16, 19, 21, 22, 24–28, 30, 34)	11 (1166)	2.60 (1.75-3.85)	45.9	0.047	4.72	<0.001
Liver metastasis (negative vs. positive) (23, 28, 34)	3 (338)	2.16 (1.31-3.54)^F^	0.0	0.555	3.04	0.002
Tumor size (small vs. large) (21, 24–28, 30, 33, 34)	9 (1012)	1.96 (1.16-3.31)	70.1	0.001	2.50	0.013
Degree of differentiation (moderate/poor vs. well) (16, 21, 24–27, 34)	7 (738)	0.68 (0.27-1.72)	86.2	<0.001	0.81	0.418

All pooled ORs were calculated from random-effect model except for cells marked with (fixed^F^). P_h_ denotes P value for heterogeneity based on Q test; P denotes P value for statistical significance based on Z test. OR: odds ratio; CI: confidence interval.

### Sensitivity analysis and publication bias

**Figure 4:**
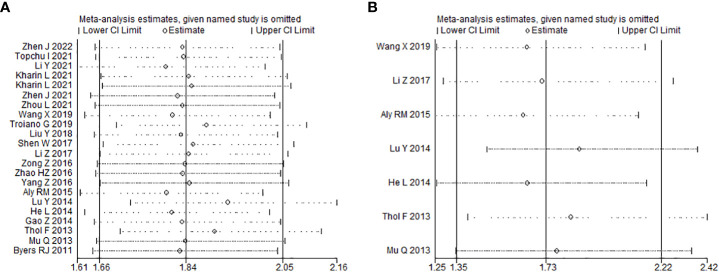
Effects of individual studies on pooled hazard ratios for MSI2 expression and survival of cancer patients. **(A)** Result of sensitivity analysis for pooled overall survival estimation. **(B)**. Result of sensitivity analysis for pooled disease-free survival estimation.

**Figure 5:**
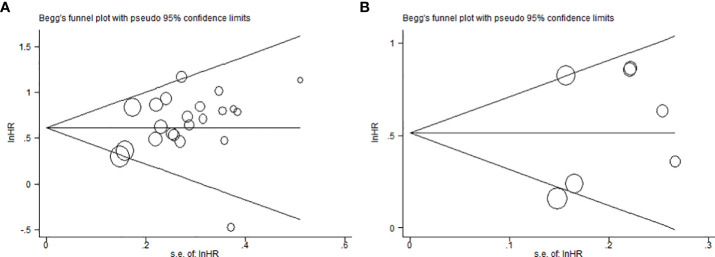
Begg’s funnel plots for assessment of potential publication bias in studies of MSI2 in cancer patients. **(A)** Funnel plot of publication bias for overall survival. **(B)** Funnel plot of publication bias for disease-free survival.

Sensitivity analysis was carried to assess the influence of each study on the meta-analysis results by omitting one study in turn. No single point estimate of the omitted individual dataset lay outside the 95% CI of the pooled analysis based on the overall HR estimate of OS (**Figure 4A**) and DFS (**Figure 4B**), indicating that the results were stable and reliable. Furthermore, all *P* values of Begg’s and Egger’s tests were greater than 0.05 (OS: Begg’s test, *P*=0.367; Egger’s test, *P*=0.168) (DFS: Begg’s test, *P*=1.000; Egger’s test, *P*=0.411), indicating that there was no publication bias in this meta-analysis. In addition, the symmetry of the funnel plots once again visually confirmed the absence of publication bias (**Figure 5A, B**).

## Discussion

MSI-2 has been shown to be involved in numerous solid and blood malignancies, and its expression is higher than in normal tissues and correlated with prognosis. However, its prognostic role in patients with cancer is inconsistent and unclear. Thus, we reviewed published literature and conducted a meta-analysis to evaluate the association between MSI2 expression and the risk of cancer mortality and relapse. Twenty-one studies including 2640 patients were included in the meta-analysis. The results demonstrated that high MSI2 expression was significantly associated with poor prognosis, with results of poor OS (HR = 1.84, 95% CI: 1.66-2.05, *P* < 0.001), and poor DFS (HR = 1.73, 95% CI: 1.35-2.22, *P* < 0.001). In addition, the association remained significant in subgroups of OS based on cancer type, detection method, sample size and analysis method. Moreover, sensitivity analysis and publication bias showed that the results were stable and reliable. Furthermore, MSI2 positive expression was associated with certain phenotypes of tumor aggressiveness. Thus, increased MSI2 expression was associated with poor survival.

The evolutionarily conserved translation regulatory protein MSI2 is a member of the Musashi family of RBP (20). It regulates mRNA translation of many intracellular targets and maintains the properties of stem cells, thereby controlling cell proliferation and differentiation (24, 44). Thus, it is widely expressed in various tumors, and the level of expression is associated with poor prognosis of the disease (8, 39). Moreover, MSI2 was identified as a metastatic driver that supported the protein expression associated with epithelial-mesenchymal transition, including E-cadherin, the tight junction protein ZO1, the cytokine TGFβ1, the small mothers against decapentaplegic homolog 3, and the zinc finger proteins SNAI1 and SNAI2 and down-regulated expression of properties-related proteins, including claudin (claudin 3, claudin 5 and claudin 7) (16, 45). Furthermore, MSI2 plays an important role in drug resistance (11, 17, 18, 46, 47). For example, increased MSI2 expression enhances resistance to epidermal growth factor receptor tyrosine kinase inhibitors that are effective in patients with NSCLC harboring EGFR mutations (17).

Based on the fact that high expression of MSI2 can predict poor prognosis in cancer patients, and the relevant regulatory mechanisms of MSI2 in tumors, therapy targeting MSI2 may have considerable potential. In addition, small molecule inhibitors of MSI2 have been shown to be effective *in vivo* or *in vitro*. Lan et al. found that Gn, a natural inhibitor of MSI1, can similarly disrupt the binding of MSI2 to Numb RNA, like MSI1, so it is considered a dual inhibitor of MSI1 and MSI2 (48). Furthermore, the use of MSI1/MSI2 dual inhibitors such as Gn in colorectal patients with MSI overexpression can achieve better efficacy (49). In addition, Lan et al. also found that Aza-9, a derivative of secondary metabolites from *Aspergillus nidulans*, is a dual Msi1/2 inhibitor that can inhibit MSI2-RNA interaction in cells (50). Moreover, Aza-9-liposome inhibits proliferation, induces apoptosis and autophagy, and down-regulates Notch and Wnt signaling in colon cancer cell lines (50). Wang et al. confirmed that the small compound largazole can bind to MSI2 and may be a potential MSI2 inhibitor (51). Largazole significantly reduces MSI2 protein and mRNA levels and inhibits its downstream mammalian rapamycin signaling pathway targets (51). Largazole also inhibits proliferation and induces apoptosis in NSCLC and chronic myeloid leukemia cells (51). Thus, the MSI2 inhibitor largazole is promising as a treatment for these malignancies. Overall, the development of MSI2 inhibitors is still in the early stage, and the development of effective and highly specific MSI2 inhibitors will provide a new strategy for precise targeted therapy of tumors (8).

Although this meta-analysis comprehensively assessed the prognostic value of MSI2 expression in cancer, some limitations should be considered. First, most of the patients included in this study were from China, which to some extent affected the applicability of the results. Second, this study only included articles in Chinese and English, missing important studies published in other languages, which resulted in a certain language bias. Third, the expression level of MSI2 was not detected by a unified method, and its grouping criteria did not adopt a consistent cut-off value, which might have some effect on the results. Fourth, several HRs were extracted from the survival curves, rather than directly obtained from the article, which can cause bias.

## Conclusion

In conclusion, the present meta-analysis demonstrated that high MSI2 expression was significantly associated with poor prognosis in various cancer patients. Thus, MSI2 can be used as a great biomarker for the prognosis of various cancers, and therapy targeting MSI2 is worthy of further study.

## Data availability statement

The data supporting this meta-analysis are from previously reported studies and datasets, which have been cited. The processed data are available within the article.

## Author contributions

Concept and design: LJ and CZ. Literature search and extracting of data: SX, JX, JW and CZ. Analyzing and interpretation of data: XF and CZ. Drafting of the manuscript: LJ. Critical revision of the manuscript: LJ and CZ. All authors listed have made a substantial, direct, and intellectual contribution to the work and approved it for publication. All authors contributed to the article and approved the submitted version.

## Funding

This work was supported by Taizhou People’s Hospital Medical Innovation Team Foundation (CXTDA201901) and Taizhou People’s Hospital Mandatory Project (ZL201929, ZL202020 and ZL202029).

## Conflict of interest

The authors declare that the research was conducted in the absence of any commercial or financial relationships that could be construed as a potential conflict of interest.

## Publisher’s note

All claims expressed in this article are solely those of the authors and do not necessarily represent those of their affiliated organizations, or those of the publisher, the editors and the reviewers. Any product that may be evaluated in this article, or claim that may be made by its manufacturer, is not guaranteed or endorsed by the publisher.

## Abbreviations

MSI2, Musashi 2; RBP, RNA-binding protein; OS, overall survival; DFS, disease-free survival; HR, hazard ratio; CI, confidence interval; IHC, immunohistochemistry; qRT-PCR, quantitative reverse transcription polymerase chain reaction; NOS, Newcastle-Ottawa Scale; OC, ovarian carcinoma; NSCLC, non-small cell lung cancer; CRC, colorectal cancer; CC, cervical cancer; PC, pancreatic cancer; HCC, hepatocellular carcinoma; OSCC, oral squamous cells carcinoma; ESCC, esophageal squamous cell carcinoma; ALL, acute lymphoblastic leukemia; GC, gastric carcinomas; AML, acute myeloid leukemia.
